# Non-parathyroid hypercalcemia in a patient with new-onset hyperthyroidism and silicone-induced granulomas: case report

**DOI:** 10.3389/fendo.2024.1447652

**Published:** 2025-01-21

**Authors:** Laura Montefusco, Giada Rossi, Iulia Petria, Ida Pastore, Paolo Fiorina

**Affiliations:** ^1^ Division of Endocrinology, Azienda Socio Sanitaria Territoriale (ASST) Fatebenefratelli-Sacco, Milan, Italy; ^2^ Dipartimento di Scienze Biomediche e Cliniche, University of Milan, Milan, Italy; ^3^ Nephrology Division, Boston Children’s Hospital, Harvard Medical School, Boston, MA, United States; ^4^ International Center for T1D, Pediatric Clinical Research Center Romeo ed Enrica Invernizzi, Dipartimento di Scienze Biomediche e Cliniche (DIBIC), University of Milan, Milan, Italy

**Keywords:** hypercalcemia, hyperthyroidism, granulomatosis, tumor-induced hypercalcemia, case report

## Abstract

**Background:**

Hypercalcemia is a frequent occurrence in hospitalized patients. It can vary in presentation and severity, and appropriate treatment requires targeting of the underlying condition. Rarer causes of hypercalcemia, such as hyperthyroidism and granulomatous diseases, need to be addressed after excluding the more prevalent etiologies, namely primary hyperparathyroidism and malignancies. We report a case of moderate hypercalcemia in a patient with HIV-positivity with new-onset autoimmune hyperthyroidism as well as concomitant chronic granulomas due to silicone injections.

**Case summary:**

A 61-year-old patient presented with generalized malaise, asthenia, dyspnea and dysphagia associated with rapid weight loss and recurrent panic attacks. Biochemical work-up revealed moderate hypercalcemia and overt hyperthyroidism with positive anti-TSH-receptor antibodies. Hydration, loop diuretics and methimazole were initiated immediately. Suppressed parathyroid hormone (PTH) levels excluded PTH-mediated hypercalcemia (e.g., primary hyperparathyroidism) and among causes of non-PTH-mediated hypercalcemia, malignancies were excluded. Granulomas secondary to past silicone injections were also found in our patient, however normal 1,25-dihydroxy vitamin D_3_ levels. Treatment of hyperthyroidism with normalization of thyroid function tests was simultaneously followed by improvement of calcium and PTH levels supporting the diagnosis of hypercalcemia secondary to Graves’ disease.

**Learning points:**

Hyperthyroidism is a rare cause of hypercalcemia, but it has to be considered in suggestive clinical settings. In our case, prompt management of Graves’ disease contributed to the normalization of calcium levels. This, in turn, supported the differential diagnosis of non-PTH-mediated hypercalcemia.

## Introduction

Hypercalcemia is a relatively common clinical condition, affecting approximately 1% of worldwide population (1). It can be classified as mild (10.5 to 11.9 mg/dL, or 2.62 to 2.97 mmol/L), moderate (12.0 to 13.9 mg/dL, or 3.0 to 3.47 mmol/L), or severe, i.e., hypercalcemic crisis (≥ 14.0 mg/dL, or ≥ 3.5 mmol/L) (2). Hypercalcemia generally underlies a clinically-significant disorder; it therefore requires investigation of potential etiologies. Approximately 90% of cases of hypercalcemia are due to primary hyperparathyroidism (PHPT) or malignancies (3). Hyperthyroidism is known to be a cause of non-parathyroid mediated hypercalcemia, with asymptomatic hypercalcemia reported in around 20% of cases of hyperthyroidism (4). Adjusted calcium levels rarely exceed 3.0 mmol/L in hyperthyroidism-related hypercalcemia and severe hypercalcemia is quite rare. To date there are only a few cases of hypercalcemia secondary to hyperthyroidism reported in literature (5–8). The mechanism of hyperthyroidism-associated hypercalcemia is not fully understood, but it has been suggested that an increase in bone turnover mediated by free triiodothyronine (FT3) may play a key role (9). Vitamin D derangements are other uncommon but important causes of hypercalcemia. Granulomatous diseases (i.e., sarcoidosis, tuberculosis, or foreign bodies granulomas) are characterized by abnormally-elevated 1,25-dihydroxy vitamin D_3_ levels [1,25-(OH)_2_-D_3_], which lead to an increase in calcium and phosphate absorption, primarily from the gut (10). We describe a case of hypercalcemia with suppressed parathyroid hormone (PTH) presenting in a patient with new-onset autoimmune hyperthyroidism and concomitant foreign-body granulomas secondary to silicone injections.

## Case presentation

**Figure 1:**
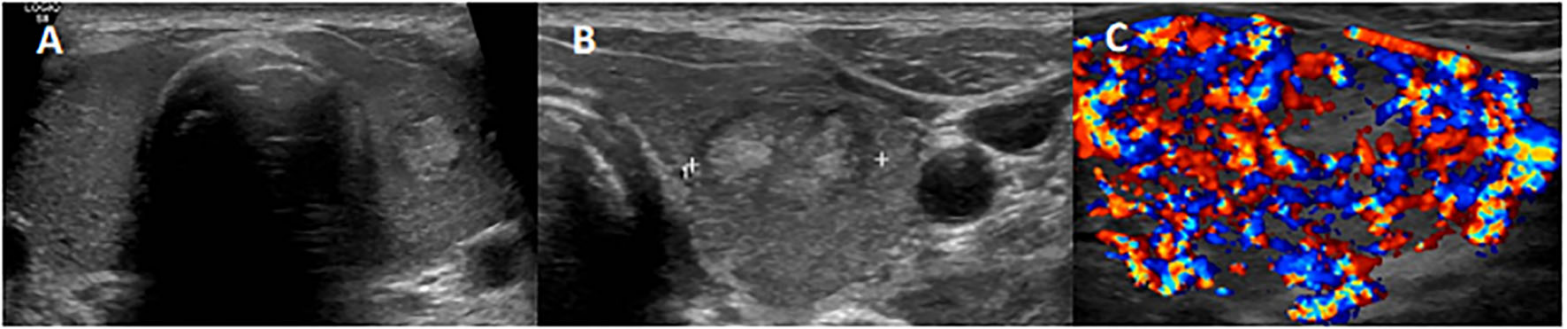
Thyroid ultrasonography (US) examination showing a non-uniform hypoechoic glandular parenchyma **(A)**, with a 17-mm well-defined hyperechoic nodule in the left lobe **(B)** and a diffused markedly accentuated vascularization as described in thyroid storm **(C)**.

**Figure 2:**
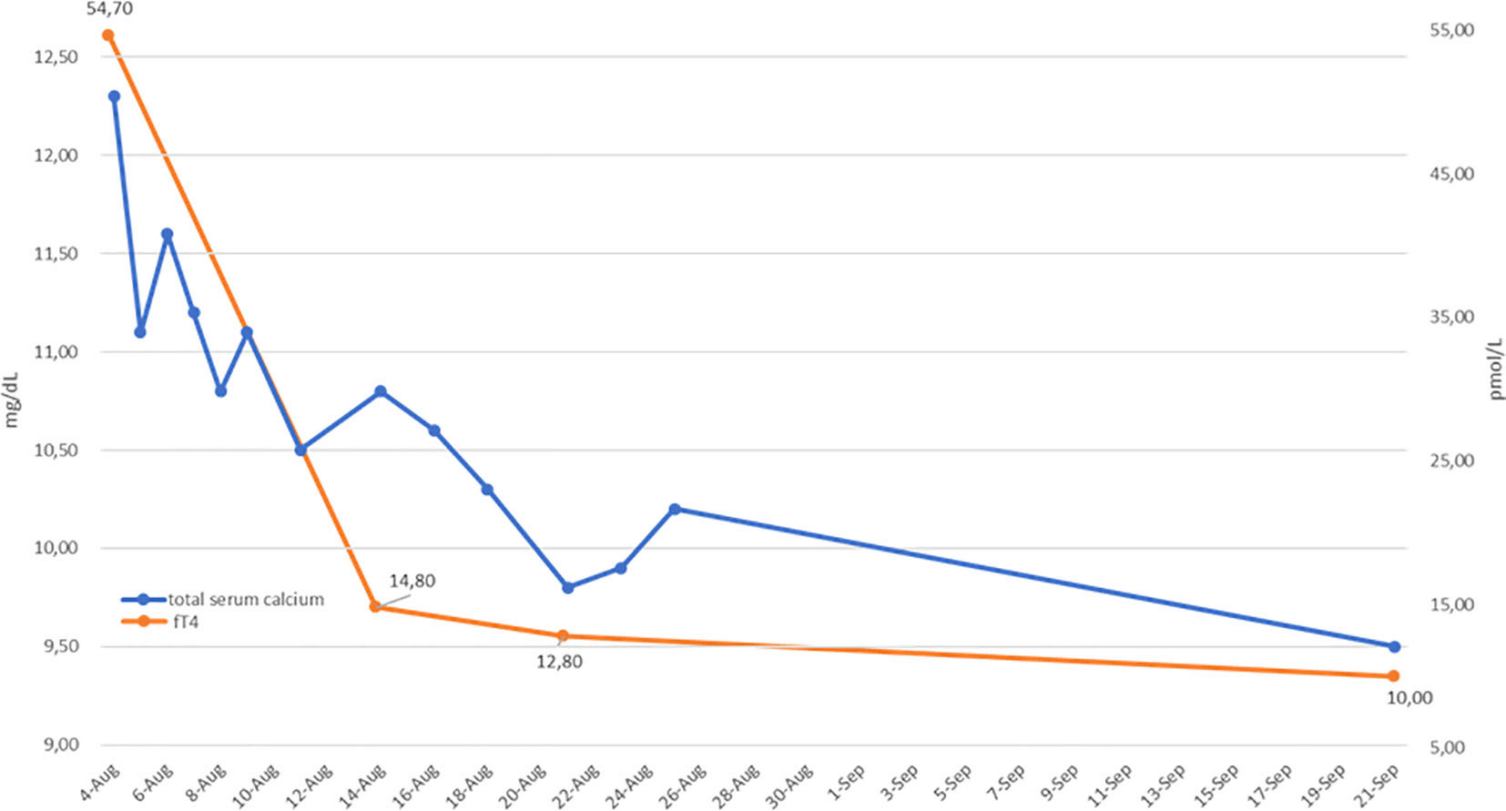
Serum adjusted total calcium (blue line) and free T4 (fT4; orange line) levels before and during hyperthyroidism treatment. A parallel reduction in fT4 and calcium values is shown.

**Figure 3:**
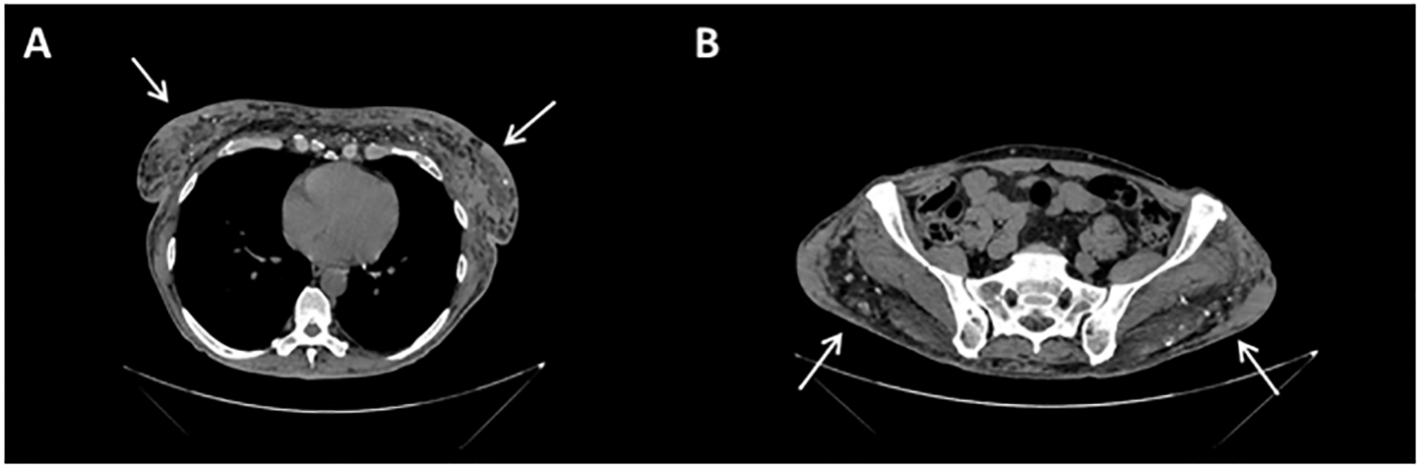
Contrast-enhanced CT scan sections: at mammary **(A)** and gluteal **(B)** level, images are compatible with patient history of silicone injections, in the form of soft-tissue densities with surrounding fat stranding (arrows) and peripheral calcifications.

A 61-year-old, assigned male at birth transgender Hispanic patient, with a history of human immunodeficiency virus (HIV) on antiretroviral therapy from 1999 (in stable control and on single tablet regimen of bictegravir, emtricitabina, tenofovir alafenamide therapy from October 2019), presented to the infectious disease clinic in July 2023. He was complaining generalized malaise, asthenia, dyspnea and dysphagia associated with weight loss of 14 kilograms over 45 days and recurrent panic attacks. He was then admitted to in-stay in the infectious disease department for further investigations. On physical examination, blood pressure was 110/65 mmHg with a heart rate of 91 beats per minute. He was afebrile and his capillary oxygen saturation was 96% in room air. Cardiovascular, respiratory, abdominal and neurological examination was unremarkable, except from some gait uncertainty. On the other hand, serum and plasma laboratory exam revealed markedly elevated adjusted total calcium levels 13.1 mg/dL, or 3.27 mmol/L (normal range: 8.5-10.5 mg/dL, or 2.12-2.62 mmol/L), suppressed TSH <0.01 mIU/mL (normal range: 0.35-4.94 mIU/mL) and significantly high free T4 (FT4) of 54.7 pmol/L (normal range: 9-19 pmol/L) and free T3 (FT3) of 26.8 pmol/L (2.4-6 pmol/L). Renal function was normal [serum creatinine 0.61 mg/dL (0.7-1.2 mg/dL), with an estimated glomerular filtration rate (eGFR) of 107.8 mL/min/1.73 m^2^], serum albumin was low (30 g/L) and liver enzymes were mildly elevated [aspartate amino transferase 45 U/L (11-34 U/L), alanine amino transferase 52 U/L (≤ 49 U/L), gamma glutamyl transferase 89 U/L (12-68 U/L)]. Alkaline phosphatase was 110 U/L (43-115), C-reactive protein was 4.5 mg/L (normal up to 10 mg/L), and blood cell count and electrolytes (sodium, potassium and chloride) were within the reference ranges. Endocrinological consultancy was asked for and hydration with saline solution (0.9% NaCl, 100 mL/h continuously) simultaneously with intravenous loop diuretic (furosemide 20 mg, twice a day) was started immediately. In order to clarify the etiology of the hypercalcemia, we recommended further biochemical and imaging exams; at the same time considering evidence of hyperthyroidism, we suggested starting with methimazole 10 mg twice a day. It has been described that anti-retroviral therapy can be associated to Grave’s Disease occurrence even several months after starting, but the patient was on stable therapy regimen for about 5 years and he never showed signs of thyroid dysfunction although he underwent regular controls in the clinic. First of all, Tc-99m thyroid scintigraphy showed an intense and homogeneous uptake throughout the entire thyroid parenchyma. Thyroid ultrasound further described an increase in thyroid volume, diffusely non-uniform structure and markedly accentuated vascularization, and a 17-mm round solid iso-hyperechoic nodule with well defined margins and predominantly peripheral vascularization in the left lobe (**Figure 1**) (11). The day after admission, total serum calcium was 11.1 mg/dL, or 2.77 mmol/L (8.5-10.5 mg/dl, or 2.12-2.62 mmol/L), ionized calcium 6.0 mg/dL (4.7-5.2 mg/dL), serum phosphate 3.0 mg/dL, or 0.97 mmol/L (2.5-4.5 mg/dL, or 0.8-1.5 mmol/L), magnesium 1.5 mg/dL (1.4-2.4 mg/dL), 25-hydroxy vitamin D_3_ (25-OH-D_3_) 50 ng/mL (20–100 ng/mL), 1,25-dihydroxy vitamin D_3_ [1,25-(OH)_2_-D_3_] 56 ng/L (20-80 ng/L), alkaline phosphatase (ALP) 101 U/L (43-115 U/L), C-terminal telopeptides of type I collagen (CTX) 1900 ng/L (120-630 ng/L), serum creatinine 0.53 mg/dL (0.7-1.2 mg/dL), 24-hour urinary calcium 20.2 mg/dL (8.3-26.6 mg/dL), and PTH was significantly low (< 5.5 ng/L; normal range 14.9-56.9 ng/L). Of note, 24-hour urinary calcium was within normal range despite commencement of diuretic treatment. In addition, anti-thyroglobulin antibodies were 69 IU/mL (TGAb, normal range: 0–115 IU/mL), anti-thyroperoxidase antibodies 476 IU/mL (TPOAb, normal range: 0–34 IU/mL), anti-TSH-receptor antibodies 20.9 IU/L (TRAb, normal range: 0–1.22 IU/L), and unstimulated calcitonin was 0.7 ng/L (up to 9.5 ng/L). Finally, serum electrophoresis revealed a IgGλ monoclonal component of 2.8 g/L. Autoimmune hyperthyroidism, or Graves’ disease, was diagnosed and methimazole was increased in dosage at 10 mg three times a day. Suppressed parathyroid hormone (PTH) levels ruled out PTH-mediated hypercalcemia (e.g., primary hyperparathyroidism) as a cause of his hypercalcemia. 25-hydroxy vitamin D values excluded vitamin D deficiency or intoxication, while 1,25-dihydroxy vitamin D levels within reference ranges made granulomatous disease-driven hypercalcemia (e.g., sarcoidosis) unlikely. Normal urinary calcium excretion was inconsistent with familial hypocalciuric hypercalcemia (FHH); at the same time, in-range alkaline phosphatase (ALP) levels were inconsistent with high-turnover bone diseases like Paget’s disease. However, the presence of monoclonal gammopathy together with low PTH suggested the presence of tumor-mediated hypercalcemia, and in particular multiple myeloma was suspected. He was therefore prescribed a whole-body computed tomography (CT) scan, with particular regard to bone segments, and additional blood analyses. No further investigations were asked for the thyroid nodule, considering its low-risk ultrasound characteristics and low calcitonin value. In the meantime, calcium levels were decreasing with hydration, diuretics combined with thyrostatic treatment (**Figure 2**; **Supplementary Table 1**), with patient’s subjective improvement in anxiety, asthenia and dysphagia. Neither bone nor ocular pain or discomfort were reported by the patient and at physical examination clinical activity score for orbitopathy was 0. Otolaryngologist examination for dysphagia was performed but normal. Contrast-enhanced total-body CT scan showed an enlarged thyroid gland, with the known nodular lesion in the left lobe but no sign of tracheal compression, some small gallstones and prostatic hypertrophy. Conversely, lungs and heart had no alterations, no pathological lymph nodes and no structural bone lesions were found. Considering the CT scan results, the modest amount of the monoclonal component (2.8 mg/dL), the absence of anemia, renal insufficiency or Bence Jones protein, and the finding of kappa free light chains (k-FLC) 44 mg/L, lambda free light chains (λ-FLC) 38.9 mg/L, with a ratio of 1.13 (0.26-1.65), lactate dehydrogenase (LDH) 122 U/L (125-220 U/L), beta-2-microglobulin 4.3 mg/L (up to 3.5 mg/L), total serum proteins 5.0 g/L (6.5-8.5 g/L), plasmatic IgG 10.45 g/L (7-16 g/L), IgA 1.93 g/L (0.7-4 g/L), IgM 0.95 g/L (0.4-2.3 g/L), the presence of multiple myeloma was excluded and Monoclonal Gammopathy of Uncertain Significance (MGUS) was diagnosed. A consultation with a hematologist was performed but no further hematological investigations were deemed necessary. Prostate cancer was also ruled out according to prostate CT characteristics and prostatic specific antigen (PSA) value within the normal range. After a week of thyrostatic treatment, intravenous hydration and diuretics, calcium levels stabilized at a level of <12 mg/dL, or <3.0 mmol/L (**Figure 2**), and thyroid function significantly improved with TSH <0.01 mIU/mL, FT4 14.8 pmol/L (9-19 pmol/L) and FT3 6.8 pmol/L (2.4-6 pmol/L). No alterations in blood count, liver enzymes and phosphate levels were observed. Heart rate (HR) and corrected QT interval (c-QT) always remained within normal limits [HR 60-100 beats per minute (bpm); c-QT 300-440 ms]. Methimazole was therefore reduced from 10 mg three times a day to 10 mg twice daily, with a parallel reduction in hydration (from saline solution 100 ml/h to saline solution 50 ml/h) and diuretics (from intravenous furosemide 20 mg twice a day to oral furosemide 25 mg daily). To complete the clinical framework, a total body 18F-fluorodeoxyglucose positron emission tomography/computed tomography (18F-FDG PET/TC) was performed. The exam highlighted homogeneous 18F-FDG uptake in the thyroid lodge and, interestingly, moderate 18F-FDG accumulation in the thoracic retro-mammary area and extensive bilateral accumulation in the gluteal regions, as per inflammatory response. The patient revealed that in the past he had silicone injections performed in those sites as part of gender transition: as a matter of fact, upon revision of previously performed CT scan sections, compatible images were visible at mammary and gluteal level (12) (**Figure 3**). No other areas of altered uptake were observed in the remaining body segments examined. Of note, no gender-affirming hormonal therapy was taken by our patient. In the following days, calcium levels continued to decrease, reaching 10.3 mg/dL (or 2.57 mmol/L) two weeks after admission; at the same time, TSH levels started to increase with a parallel decrease in free thyroid hormone fractions (TSH 0.05 mIU/mL, FT4 12.8 pmol/L, FT3 4.1 pmol/L). Intravenous hydration was suspended and replaced by oral hydration, while furosemide (25 mg daily) and methimazole (10 mg twice daily) were continued and the patient was discharged from hospital. At discharge patient was not complaining dysphagia or dyspnea any more, he referred amelioration of weakness and anxiety and a general sense of improved well-being. However, PTH levels still remained suppressed (<5.5 ng/L), thus, at the time of discharge, it was not possible to distinguish between hyperthyroidism-related hypercalcemia and chronic granulomatosis-related hypercalcemia due to foreign-body (silicone injections). PTH trend after thyroid function stabilization to normal ranges was particularly helpful to clarify the etiology of hypercalcemia. In fact, three weeks after hospital discharge, both thyroid function and calcium serum levers further improved (TSH <0.01 mIU/mL, FT4 10 pmol/L, FT3 5.4 pmol/L, adjusted total serum calcium 10.0 mg/dL or 2.5 mmol/L) and PTH levels re-established to normal values (PTH 21 ng/L; normal range 14.9-56.9 ng/L), which made hypercalcemia secondary to hyperthyroidism the most likely diagnosis in the case of our patient. After four months of methimazole therapy anti-TSH-receptor antibodies were still high (37.9 IU/L, normal range: 0–1.22 IU/L), with free thyroid hormones in normal range and a still suppressed TSH. At follow up a bone mineral density study was performed and it excluded osteoporosis (Spine L1-L4 T-score -0.1, Femoral neck T-score -1.1, Femoral total T-score -0.7, performed with Lunar instrument) while CTX levels decreased to 430 ng/L (120-630 ng/L) despite no use of any anti-osteoporotic treatment.

## Discussion

We presented a case of moderate hypercalcemia in a patient affected by HIV-positivity with new-onset autoimmune hyperthyroidism as well as concomitant granulomas due to foreign bodies (silicone injections). In general, hypercalcemia can vary in presentation and severity, ranging from asymptomatic to a life-threatening emergency. A retrospective study by Nongnuch et al. (13) showed that most people living with HIV developing hypercalcemia were asymptomatic, and suggested that corrected serum calcium of ≥12 mg/dL should be investigated for underlying solid organ malignancy. In fact, in people living with HIV, major causes of hypercalcemia include solid organ malignancy, hematologic malignancy, and infections. In the general population the most common causes of hypercalcemia are primary hyperparathyroidism (PHPT) and malignancy; PTH is elevated in PHPT while suppressed in malignancy-related hypercalcemia (2). Other non-PTH-mediated etiologies of hypercalcemia include endocrine disorders, granulomatous diseases, and drugs (14). In the case we illustrated, hyperthyroidism was a possible cause of PTH-suppressed hypercalcemia, after exclusion of tumor- and granulomatosis-related hypercalcemia. As regards hypercalcemia secondary to neoplasm, underlying mechanisms vary by cancer type: in primary malignancies (e.g., lung, breast, kidney, skin) by producing parathyroid hormone-related protein (PTHrP) (14), in secondary malignancies (i.e., skeletal metastases) and myeloma by focal increases in bone resorption (15) in some instances of lymphomas by abnormal synthesis of 1,25-(OH)_2_-D_3_ in tumor-associated macrophages (16) and finally rarely by ectopic PTH secreted by certain malignant tumors (1). In the case we presented, solid malignancies were excluded basing on total-body imaging, and the presence of monoclonal gammopathy prompted further hematological investigations that ruled out multiple myeloma. With reference to granulomatosis-related hypercalcemia, the main pathological mechanism is the overexpression of 1-α-hydroxylase in macrophages, which are a main cell type involved in the granuloma reaction. Efficacy in the regulation of vitamin D metabolism, however, is impaired in macrophages, with production of 1-α-hydroxylase being unresponsive to feedback by activated vitamin D3, unlike in proximal kidney tubule cells, where 1-α-hydroxylase production is limited by levels of calcitriol. This leads to abnormally increased levels of 1,25-dihydroxy-vitamin D in granulomatous diseases, which in turn, increases blood calcium levels (17). Liquid or injectable silicone is a non-biodegradable material used in the past as an injectable filler for cosmetic body contouring. Although previously considered inert, silicone may generate inflammatory reactions, with granuloma formation being a rare but well-described complication. Thus, injectable silicone can lead to non-PTH mediated hypercalcemia through granuloma generation (3, 17–19). In our patient, a PET/TC scan revealed moderate 18F-FDG uptake in the retro-mammary area and extensive accumulation in the gluteal region, as per granulomatous inflammatory response to old silicone injections. However, 1,25-(OH)_2_-D_3_ levels within reference range and the rise in PTH values after discharge from hospital and thyroid function normalization made granulomatosis-related hypercalcemia unlikely. In people living with HIV other granulomatosis-related conditions inducing increased vitamin D hydroxylation such as tuberculosis should be excluded (13). Coming to hyperthyroidism-related hypercalcemia, the most supported explanation is increased bone turnover mediated by free thyroid hormones, with transfer of calcium from bone to serum. High levels of bone markers, such as C-terminal telopeptides of type I collagen (CTX) and alkaline phosphatase (ALP), were reported in hyperthyroid patients (20) similarly to what observed in our case report and their evaluation resulted helpful in characterizing the condition described. Although intestine and kidneys attempt to prevent calcium accumulation with decreased re-absorption and increased excretion respectively, fT3-stimulated osteoclast activation due to thyrotoxicosis results in overall increase of calcium serum levels (14). Histologic and morphometric bone changes described in bone biopsies before and after treatment for hyperthyroidism are consistent with sustained bone turnover (20). Another mechanism of hypercalcemia in Grave’s disease may involve PTHrP: a positive correlation between increased PTHrP levels and ionized calcium was observed in untreated hyperthyroid patients with simultaneously decreased levels of PTH (21). In our clinical case, we described how prompt initiation of methimazole treatment in hyperthyroidism-related hypercalcemia led to normalization of both serum calcium and PTH levels as soon as normal thyroid function was achieved. This probably also restored normal bone turnover, so that in our case there was no need for adjunctive therapies, such as bisphosphonates. Of course, diagnosis of hyperthyroidism-related hypercalcemia can only be reached after exclusion of other etiologies, but the excellent response to treatment strongly supports hyperthyroidism-related hypercalcemia as the most likely diagnosis.

The cornerstone of immediate management of hypercalcemia is intravenous rehydration, generally with normal saline solution, in order to increase glomerular filtration and calcium excretion (14). Diuretics can be added to prevent fluid overload, however their effect on calcium excretion is limited (22). Furthermore, bisphosphonates or calcitonin can be added to the treatment of moderate-severe hypercalcemia or in very symptomatic patients (2). It is likewise fundamental to target the cause of hypercalcemia (19, 22). For instance, thyrostatic agents such as thionamides (e.g., carbimazole, methimazole, propylthiouracil) are the primary treatment for hypercalcemia associated with hyperthyroidism (14). A literature search with PubMed research criteria: “hyperthyroidism AND Graves AND hypercalcemia AND case report” produces 34 results. Among those 13 papers have been published before 2000, 4 papers were not published in English or in international journals and 3 papers described different conditions (i.e. calcific uremic arteriopathy, membraneous nephropathy and polyuria) and excluded. The remaining 14 results of case report that already describedthe management and outcomes of Graves’ hyperthyroidism and hypercalcemia with or without other concomitant conditions are summarized in **Table 1**. In our case, intravenous hydration combined with diuretics and methimazole successfully controlled thyroid status as well as serum calcium levels.

**Table 1:** Summary of case reports of Graves’ Disease and Hypercalcemia, with treatment and outcomes (PubMed research criteria: “hyperthyroidism AND Graves AND hypercalcemia AND case report”, inclusion criteria: published in English, in international journals, after the year 2000).

First Author	Journal	Year	Primary and Concomitant Conditions	Hypercalcemia Severity^a^	Treatment	Outcome(s)
Giovanella L, et al. (23)	New England Journal of Medicine	2008	Graves’ Disease, Thymus Enlargement	Moderate	Carbimazole	Normalization of thyroid function and calcium levels, shrinkage of the thymic mass
Korytnaya E, et al. (24)	Clinical Medicine Insights: Endocrinology and Diabetes	2011	Graves’ Disease, Vitamin D Deficiency	Mild	Methimazole, Atenolol, subsequent Radioiodine, Vitamin D Supplementation	Normalization of thyroid function and calcium levels
Yokomoto M, et al. (25)	Internal Medicine	2015	Graves’ Disease, Primary Hyperparathyroidism, Vitamin D Deficiency	Severe (Hypercalcemic Crisis)	Methimazole, Isotonic Saline, Furosemide, Hydrocortisone, Calcitonin, Potassium Iodide, subsequently Oral Cinacalcet	Normalization of calcium levels only after intravenous bisphosphonates and thyro-parathyroidectomy
Gupta K, et al. (26)	Clinical Case Reports	2017	Graves’ Disease	Moderate	Metoprolol, Carbimazole, Intravenous Fluids and Loop Diuretics	Moderate hypercalcemia persisted, therefore Pamidronate was administered, with subsequent hypocalcemia managed with calcium supplementation and calcitriol
Baldane S, et al. (27)	Endocrine Regulations	2016	Graves’ Disease, Primary Hyperparathyroidism	Severe (Hypercalcemic Crisis)	Methimazole, Isotonic Saline, Furosemide, Calcitonin, Zoledronic Acid, subsequently Thyroidectomy and Right Inferior Parathyroid Gland Adenoma Excision	Normalization of calcium levels and thyroid function with medical treatment; resolution of primary conditions after surgical treatment
Chen K, et al. (6)	Medicine	2017	Graves’ Disease	Severe (Hypercalcemic Crisis)	Methimazole, Propranolol, Physiological Saline, Furosemide, Salmon Calcitonin	Resolution of hyperthyroidism and hypercalcemic crisis
Muller I, et al. (14)	British Medical Journal	2018	Graves’ Disease	Moderate	Carbimazole, Physiological Saline, Pamidronate	Normalization of calcium levels and liver enzymes; inadequate control of hyperthyroidism, therefore total thyroidectomy
Uchida T, et al. (28)	Endocrine Journal	2020	Graves’ Disease, Breast Cancer	Severe (Hypercalcemic Crisis)	Thiamazole and Potassium Iodine for Hyperthyroidism, Discontinuation of the Aromatase Inhibitor that was suspected of causing Hypercalcemia, Saline Infusion, and Calcitonin	Hyperthyroidism improved, but the hypercalcemia persisted for one month; subsequent administration of zoledronic acid improved the hypercalcemia
Bhat S and Davis S (29)	AACE Clinical Case Reports	2019	Graves’ Disease, Primary Hyperparathyroidism due to Multiple Endocrine Neoplasia Type 1	Moderate	Subtotal Thyroidectomy and Total Parathyroidectomy with Forearm Autotransplantation	Normalization of calcium levels
Yan D, et al. (30)	BMC Endocrine Disorders	2020	Graves’ Disease, Thymus Enlargement	Moderate	Methimazole, Propranolol	Normalization of thyroid function and calcium levels, shrinkage of the thymic mass
Khan H, et al. (5)	British Medical Journal Case Reports	2021	Graves’ Disease	Severe (Hypercalcemic Crisis)	Propylthiouracil, Oral and Intravenous Rehydration, Furosemide, Pamidronate	Mild hypocalcemia and euthyroidism
Wei XD, et al. (8)	Journal of International Medical Research	2022	Graves’ Disease, Recurrent Vomiting	Mild	Methimazole, Propranolol, Fluid Replacement, Furosemide, Nutritional Support, Potassium Supplementation	Resolution of hypercalcemia, gradual resolution of hyperthyroidism and vomiting
Kaur K, et al. (31)	British Medical Journal Case Reports	2022	Intractable Vomiting	Moderate	Intravenous Hydration Carbimazole, Propanonol	Normalization of calcemia
Ajwah I, et al. (7)	Cureus	2023	Graves’ Disease, Jaundice	Moderate	Methimazole, Atenolol, Isotonic Saline, Calcitonin, Intravenous Potassium	Normalization of calcium and potassium levels and reduction in bilirubin concentration

a Mild (10.5 to 11.9 mg/dL, or 2.62 to 2.97 mmol/L), moderate (12.0 to 13.9 mg/dL, or 3.0 to 3.47 mmol/L), severe, i.e., hypercalcemic crisis (≥ 14.0 mg/dL, or ≥ 3.5 mmol/L).

## Conclusion

In summary, we presented a case of moderate hypercalcemia in a patient with new-onset autoimmune hyperthyroidism and concomitant granulomas secondary to foreign bodies (silicone injections). Differential diagnosis between the two likely etiologies (thyroid disease and granulomatosis) was endorsed by normal levels of 1,25-(OH)_2_-D_3_, the trend in PTH levels, and the improvement of calcemia in parallel with normalization of thyroid function. Such causes of hypercalcemia, although rare, need to be considered under particular circumstances, as proper etiological diagnosis is crucial to address the correct clinical management of hypercalcemia.

## Patient perspective

‘Before hospitalization I did not feel like myself anymore. I lost almost 15 kilograms in less than two months and gradually feeling worse and discombobulated I started developing terrifying panic attacks, which prevented me from living my life as I used to. My dear ones were very concerned. After starting medications, I regained my strength, appetite and my mood improved day after day. I really want to thank all the medical team for having promptly recognized the cause of my discomfort’.

## Data Availability

The original contributions presented in the study are included in the article/**Supplementary Material**. Further inquiries can be directed to the corresponding author.
