# Supplementary material for: Non-parathyroid hypercalcemia in a patient with new-onset hyperthyroidism and silicone-induced granulomas: case report
Source: Front Endocrinol (Lausanne). 2025 Jan 21;15:1447652. doi: 10.3389/fendo.2024.1447652 (PMC11790437; doi:10.3389/fendo.2024.1447652)
Supplement: Supplementary file 1 [file Table1.docx]

**Supplementary Table 1.** Patient’s exams at baseline and at follow-up days.

| Analyte | Normal range | Admission | Day 1 | Day 2 | Day 3 | Day 4 | Day 5 | Day 7 | Day 10 | Day 12 | Day 14 | Day 17 | Day 19 | Day 21 | Day 46 |
| --- | --- | --- | --- | --- | --- | --- | --- | --- | --- | --- | --- | --- | --- | --- | --- |
| Albumin adjusted-total Calcium (mg/dL) | 8.5-10.5 | 13.1 |  |  |  | 11.7 |  | 11.5 | 11.8 | 11.4 | 11.2 | 10.5 | 10.6 | 10.8 | 10.0 |
| Total Serum Calcium (mg/dL) | 8.5-10.5 | 12.3 | 11.1 | 11.6 | 11.2 | 10.8 | 11.1 | 10.5 | 10.8 | 10.6 | 10.3 | 9.8 | 9.9 | 10.2 | 9.5 |
| Ionized Calcium (mg/dL) | 4.7-5.2 |  |  | 6.0 |  |  |  |  |  |  |  |  |  |  |  |
| FT4 (pmol/L) | 9.0-19.0 | 54.7 |  |  |  |  |  | 14.8 |  |  | 12.8 |  |  |  |  |
| Albumin (g/L) | 35-50 | 30 |  |  |  | 29 |  | 27 | 28 | 30 | 29 | 31 | 31 | 33 | 34 |

Legend to Table S1 : FT4, free thyroxine.
